# Nanovolcano microelectrode arrays: toward long-term on-demand registration of transmembrane action potentials by controlled electroporation

**DOI:** 10.1038/s41378-020-0178-7

**Published:** 2020-08-24

**Authors:** Benoît X. E. Desbiolles, Etienne de Coulon, Nicolas Maïno, Arnaud Bertsch, Stephan Rohr, Philippe Renaud

**Affiliations:** 1 Laboratory of Microsystems LMIS4, Ecole Polytechnique Fédérale de Lausanne, Lausanne, Switzerland; 2 Department of Physiology, Laboratory of Cellular Optics II, University of Bern, Bern, Switzerland

**Keywords:** Biosensors, Nanofabrication and nanopatterning

## Abstract

Volcano-shaped microelectrodes (nanovolcanoes) functionalized with nanopatterned self-assembled monolayers have recently been demonstrated to report cardiomyocyte action potentials after gaining spontaneous intracellular access. These nanovolcanoes exhibit recording characteristics similar to those of state-of-the-art micro-nanoelectrode arrays that use electroporation as an insertion mechanism. In this study, we investigated whether the use of electroporation improves the performance of nanovolcano arrays in terms of action potential amplitudes, recording durations, and yield. Experiments with neonatal rat cardiomyocyte monolayers grown on nanovolcano arrays demonstrated that electroporation pulses with characteristics derived from analytical models increased the efficiency of nanovolcano recordings, as they enabled multiple on-demand registration of intracellular action potentials with amplitudes as high as 62 mV and parallel recordings in up to ~76% of the available channels. The performance of nanovolcanoes showed no dependence on the presence of functionalized nanopatterns, indicating that the tip geometry itself is instrumental for establishing a tight seal at the cell–electrode interface, which ultimately determines the quality of recordings. Importantly, the use of electroporation permitted the recording of attenuated cardiomyocyte action potentials during consecutive days at identical sites, indicating that nanovolcano recordings are nondestructive and permit long-term on-demand recordings from excitable cardiac tissues. Apart from demonstrating that less complex manufacturing processes can be used for next-generation nanovolcano arrays, the finding that the devices are suitable for performing on-demand recordings of electrical activity from multiple sites of excitable cardiac tissues over extended periods of time opens the possibility of using the devices not only in basic research but also in the context of comprehensive drug testing.

## A shockingly simple solution for electrophysiology

Arrays of microelectrodes allow researchers to record electrophysiological data from healthy, living cells for extended durations. Such measurements, which can offer valuable insights into phenomena like neuronal firing or muscle excitation, are typically cumbersome to collect. Researchers led by Benoit Desbiolles of the École Polytechnique Fédérale de Lausanne in Switzerland have devised a promising alternative, based on so-called ‘nanovolcanoes’. These hollow, conical electrodes form a seal with cell membranes. By then applying a controlled electric field across the membrane, the researchers opened up transient pores in the membrane, permitting the recording of membrane voltage changes. This enabled them to reliably monitor the electrophysiological activity of more than a dozen cells in parallel with a single device and to perform transmembrane voltage recordings at will over the course of a two-day period without injuring the cells.

## Introduction

Cell membrane electroporation is a well-established method for gaining access to the cell interior. This technique is based on the application of voltage pulses of sufficient magnitude that cause dielectric breakdown of the cell membrane, thereby inducing the formation of nanopores^1,2^. Over the past decades, biological and medical applications of electroporation^3,4^ have mainly focused on therapeutic gene transfer^5–7^, extraction of molecules^8–10^, and electrochemotherapy^11–13^. More recently, electroporation has become a method of choice for imparting micro-nanoelectrodes with intracellular access capabilities. In the context of nanoelectrophysiology, electroporation combined with multielectrode arrays (MEAs) has the potential to overcome some limitations of the current gold standard, i.e., whole-cell patch-clamp recording. This technology offers only limited throughput, the recording duration is generally short, and performing experiments is associated with high personnel costs^14^. By contrast, the use of electroporation in conjunction with arrays of nanoneedle-like electrodes recently enabled simultaneous recordings of intracellular action potentials (APs) from hundreds of electrotonically coupled rat cardiomyocytes over several days^15–17^ as well as from thousands of neurons^18^. Recordings made with electrodes of various geometries such as micromushrooms^19–22^ or nanotubes^23^ demonstrated similar electrophysiological characteristics. Current limitations of these technologies include substantial signal attenuation, short intracellular recording durations, and low yield.

In our recent work, we described a novel type of nanopatterned volcano-shaped hollow microelectrode called a “nanovolcano,” which is suited to perform attenuated transmembrane voltage measurements from networks of cardiomyocytes without the need for active electroporation^24^. The inner surface of the slightly conical hollow nanovolcanoes having a diameter of 2 μm consists of a large three-dimensional Pt electrode that offers low-impedance access to the cell interior. The outer surface of the nanovolcano is insulated by a 50-nm-thick SiO_2_ layer. A 10–20-nm-thick gold layer flanked by two Ti layers is intercalated between the Pt and SiO_2_ layers that form the nanovolcano wall. The gold layer is exposed along the upper rim of the nanovolcano and is functionalized with alkanethiol self-assembled monolayers^25^. This functionalization has been previously shown in planar electrodes to support the formation of gigaohm seals lasting several days^26^. Further facilitating seal formation, as previously reported for nanopillars^27,28^, the 100-nm-thin upper rim of the nanovolcano induces high-curvature regions in the membrane of contacting cells, which are expected to maximize the coupling between the cell and the electrode.

We have shown that nanovolcanoes permit the recording of attenuated transmembrane APs from spontaneously active monolayer cultures of primary neonatal rat cardiomyocyte cultures without applying active electroporation^24^. The yield of AP recordings with amplitudes of 6.6 ± 1.8 mV (*N* = 3087) was 15 ± 11% (*N* = 4), and continuous recordings for 18.2 ± 21 min (*N* = 16) were achieved. Static charge-induced electroporation was suspected to be the main mechanism responsible for the spontaneously occurring sporadic intracellular access of nanovolcanoes. If this hypothesis is correct, controlled electroporation applied to all nanovolcanoes of the array should improve the yield of electrodes showing intracellular access. Moreover, controlled electroporation may improve the amplitudes of the recorded signals by reducing the access resistance and may permit longer duration measurements. Hypothetically, the quality of recordings may furthermore benefit from the thiol functionalization of the gold ring that is in contact with the cells.

In the present study, we investigated these questions using spontaneously active monolayer cultures of neonatal rat ventricular cardiomyocytes^29^. Optimal electroporation waveforms and frequencies were derived from a theoretical model of the cell–electrode interface. The usefulness of thiol functionalization was tested by comparing the performance of nanovolcanoes produced with and without this feature. Controlled electroporation with nanovolcano arrays enabled on-demand intermittent intracellular access for 2 days, with recorded AP amplitudes (APAs) reaching 62 mV and yields as high as ~76%. Nonpatterned nanovolcanoes showed similar performance compared with that of nanovolcanoes containing functionalized gold nanorings.

## Results

### Electroporation modeling

**Fig. 1:**
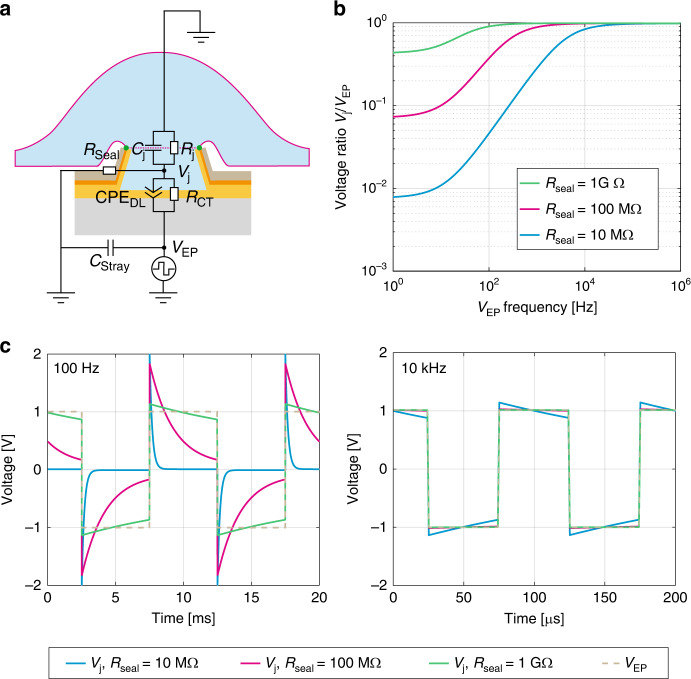
Electroporation model. **a** Equivalent electrical circuit of the cell–nanovolcano interface during electroporation. CPE_DL_ is a constant phase element representing the double layer at the electrode–electrolyte interface, *R*_CT_ is the charge-transfer resistance reflecting the faradaic charge-transfer at the same interface, *C*_*j*_ and *R*_*j*_ model the junctional capacitance and resistance of the cell membrane, respectively, *R*_Seal_ is the seal resistance at the cell–electrode interface, and *C*_Stray_ represents the capacitive current leaks along the electrical tracks. **b** Frequency dependence of the junctional voltage amplitude ratio (*V*_*j*_/*V*_EP_) of an electroporation pulse for different seal resistances (*R*_seal_). **c***V*_*j*_ waveforms in response to square biphasic electroporation pulses (*V*_EP_, stippled line) applied at 100 Hz (left panel) and 10 kHz (right panel) for different values of *R*_seal_ (blue: 10 MΩ; red: 100 MΩ; green: 1 GΩ)

A theoretical model representing the cell–electrode interface was established to analytically determine the optimal voltage waveform and frequency necessary to reliably electroporate cell membranes with nanovolcano electrodes. Figure 1a illustrates the electrical equivalent circuit of the cell–nanovolcano interface. The electrode–electrolyte interface is composed of a nonlinear resistance, *R*_CT_, that represents faradaic charge-transfer secondary to redox reactions, in parallel with a constant phase element, CPE_DL_, that represents the double layer formed by the accumulation of opposite charges at the electrode–electrolyte interface underlying capacitive charge-transfer. The stray capacitance, *C*_Stray_, denotes the capacitive current leaks along the insulated conductive tracks. A complete electrochemical characterization of the electrode–electrolyte interface was described in detail in our previous work^24^. The junctional cell membrane covering the microelectrode is modeled by its junctional capacitance, *C*_*j*_, and the parallel resistance, *R*_*j*_. The nonjunctional membrane impedance is neglected. The seal resistance, *R*_Seal_, depicts the current leaks at the cell–electrode interface. To simplify the model and consider the worst-case scenario, the resting membrane potential of the cell was not taken into account. Accordingly, *V*_*j*_ directly reflects the potential applied across the cell membrane.

To induce dielectric breakdown of cell membranes, the junctional potential across the cell membrane (*V*_*j*_) must reach 300–400 mV^30,31^. Due to the voltage divider formed by the electrode impedance at the electrode–electrolyte interface (*R*_CT_ || CPE_DL_) and the impedance representing the remainder of the cell–electrode interface (*R*_Seal_ || *C*_*j*_ || *R*_*j*_), *V*_*j*_ is attenuated compared with the electroporation voltage (*V*_EP_) applied at the terminals of the device. This attenuation should be minimized to keep *V*_EP_ low and prevent local hydrolysis in the junctional space. Figure 1b depicts the evolution of the attenuation ratio *V*_*j*_/*V*_EP_ as a function of the frequency of the sinusoidal electroporation signal applied at different values of *R*_Seal_ ranging from 10 MΩ to 1 GΩ. At low frequency, the voltage divider determined by *R*_Seal_ dominates attenuation because the junctional impedance, especially *R*_*j*_, is much higher. Therefore, an electroporation signal oscillating at 1 Hz is attenuated by a factor of 128 if *R*_Seal_ = 10 MΩ or by a factor of 2 if *R*_Seal_ = 1 GΩ. With increasing *V*_EP_ frequency, the attenuation factor decreases because the double-layer impedance (*Z*_CPE,DL_) and junctional cell membrane impedance (*Z*_Cj_) decrease. At sufficiently high frequencies, *C*_*j*_ replaces *R*_Seal_ as the dominant factor, and because CPE_DL_ is larger than *C*_*j*_, no attenuation is present anymore. Details regarding the analytical computations can be found in the Supplementary Information, Section 2.

Figure 1c shows the shape of 100 Hz and 10 kHz biphasic square electroporation pulses with amplitude *V*_EP_ together with *V*_*j*_ induced across the cell membrane. As opposed to sinusoidal waves, biphasic square waves have the distinct advantage of reaching electroporation-relevant levels of *V*_j_ nearly instantaneously and then maintaining these levels during most of the signal period. At 100 Hz and *R*_Seal_ < 1 GΩ, high-frequency components of *V*_EP_ are efficiently transmitted to the junctional space, whereas the lower-frequency parts are drastically attenuated. At 10 kHz, the cell membrane voltage follows *V*_EP_ with high fidelity even for low seal resistances, thereby ensuring a more efficient electroporation process for a similar overall pulse duration. According to this finding, in this study, we used 10 kHz biphasic electroporation voltage pulses with an overall train duration of 1 s. Higher frequencies were avoided to prevent current leaks through the stray capacitance.

### Influence of the functionalized nanopattern on electrophysiological recordings made with a nanovolcano array

**Fig. 2:**
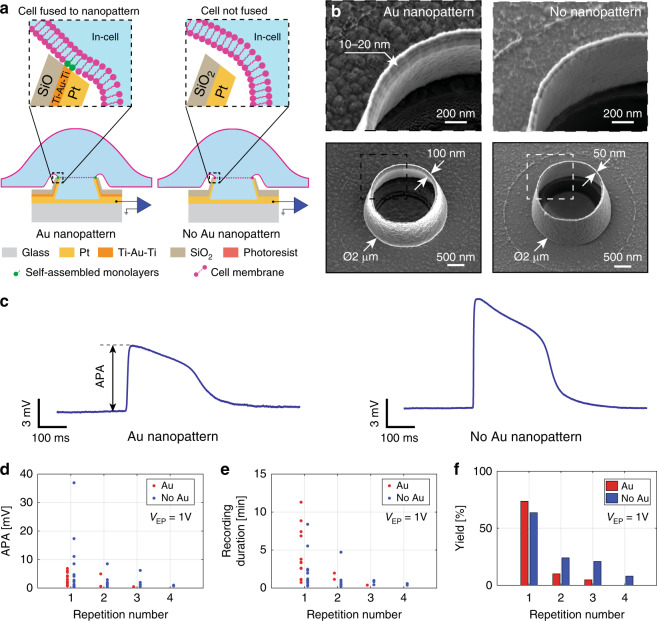
Effect of functionalized Au nanopatterns on nanovolcano performance. **a** Schematic drawings showing the overall structure of a nanovolcano with (left panel) or without (right panel) a functionalized Au nanopattern in contact with the cell. The insets depict the schematic configuration of the respective cell–electrode interfaces. The electroporated junctional cell membrane is represented by dashed lines. **b** SEM images of the nanovolcano with (left panels) and without (right panels) the Au nanopattern. The expanded views show the nanowall rims of both types of nanovolcanoes. **c** Cardiomyocyte action potentials recorded using nanovolcanoes with (left panel) and without (right panel) the Au nanopattern (APA action potential amplitude). **d** Evolution of APAs during consecutive electroporations (each data point represents the average of the first three APAs recorded by different nanovolcanoes with intracellular access). **e** Intracellular recording durations per nanovolcano as a function of consecutive electroporations. **f** Dependence of the yield (number of nanovolcanoes showing in-cell APs/total number of nonsaturated nanovolcanoes per device) on the number of consecutive electroporations

Two features of the volcano-shaped microelectrodes likely determine the quality of the electrophysiological recordings: (a) the high curvature formed by the rim of the nanovolcano is thought to force the cell membrane to follow its shape, thereby increasing the contact area, and (b) the self-assembled alkanethiol monolayer on the gold nanoring exposed on top of the nanovolcano is hypothesized to support tight adhesion of the cell membrane. Both of these features are expected to increase *R*_Seal_ and, hence, raise the signal amplitude. Here, we investigated the relative importance of the two features for establishing a high *R*_Seal_. For this purpose, nanovolcanoes with and without thiol-functionalized gold nanorings were produced, as shown schematically in Fig. 2a and by scanning electron micrographs in Fig. 2b. The fabrication process of nanopatterned volcanoes is described in detail in our previous publication^24^. Both types of nanovolcanoes have similar geometries, except for thinner walls in the case of nanovolcanoes that are devoid of a Ti–Au–Ti layer (50 nm instead of 100 nm). Each nanovolcano array was composed of 28 recording sites that were laid out according to the schematic drawing shown in the Supplementary Information, Section 1.

The experimental protocol used to compare the performance of the two types of nanovolcanoes consisted of simultaneously applying electroporation pules as defined above (1 V peak-to-peak amplitude, 10 kHz frequency, 1 s duration) to every microelectrode of devices covered with a monolayer of spontaneously active cardiomyocytes. A phase contrast image of a representative culture of a neonatal rat cardiomyocyte monolayer grown on a nanovolcano array is shown in the Supplementary Information, Section 4. The efficacy of electroporation was assessed by determining the APA, the recording duration, and the yield. A new electroporation was started when all nanovolcanoes of a given device had lost their signals, which resulted in sequential electroporations separated by 5–45 min. Typical examples of successful intracellular recordings of APs obtained by either type of nanovolcano are shown in Fig. 2c. The electrophysiological characteristics presented in the study were extracted from such traces, with the selection criteria defined in the “Methods” section.

An overall analysis of APAs recorded immediately after the given electroporations is presented in Fig. 2d for a device with and without functionalized gold rings. Each data point represents the average of APAs of the first three APs recorded by nanovolcanoes with intracellular access. Following the first electroporation (repetition number 1), each type of device reported intracellular activity in 14 channels (yield_Au_: 73.7%, yield_NoAu_: 63.6%) with APAs ranging from 0.8 to 6.8 mV (3 ± 2.1 mV, *N* = 14; nanopatterned) and 0.3 to 36.9 mV (6.6 ± 10.1 mV, *N* = 14; nonpatterned). Signals recorded by all nanovolcanoes with intracellular access declined over time, with a typical example shown in the Supplementary Information, Section 3. After intracellular signals were lost in all channels of a given device, electroporation was repeated (repetition number 2 to 4). APAs as well as the number of channels showing in-cell activity decreased with each further electroporation. After four consecutive electroporation events, the nanopatterned device did not show intracellular activity anymore, whereas in the case of nonpatterned volcanoes, two channels continued to show activity, albeit with very small APAs (<1 mV). Recording durations, i.e., the time until signals completely disappeared, are presented in Fig. 2e. Following the initial electroporation, the recording durations ranged from 44 s to 11.3 min (4.1 ± 3.3 min, *N* = 14; patterned devices) and from 16 s to 8.4 min (2.1 ± 2.2 min, *N* = 14; nonpatterned devices). With each further electroporation, the recording durations decreased. Figure 2f shows the intracellular access yield as defined by the number of channels showing intracellular activity divided by the total number of nonsaturated channels per device. Similar yields were observed after the first electroporation for both patterned (73.7%) and nonpatterned (63.6%) devices. As was the case for APAs and recording durations, the yield decreased with each consecutive electroporation.

The results indicate that nonpatterned nanovolcanoes perform similarly to those with thiol-functionalized gold rings. This finding suggests that the nanovolcano geometry is the dominant parameter in establishing conditions favorable for obtaining intracellular access. For this reason, only nonpatterned devices were used in the remainder of the study.

### Effect of the electroporation voltage amplitude on the quality of electrophysiological measurements

**Fig. 3:**
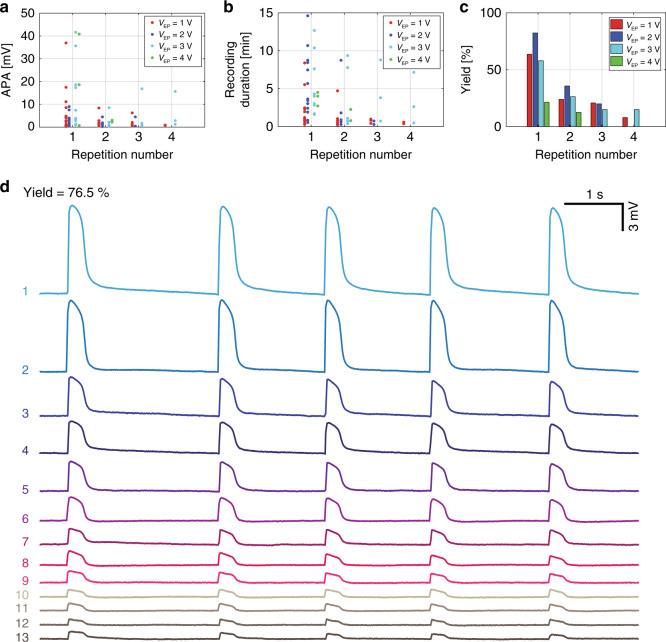
Effect of electroporation voltage amplitude (VEP) on the success of intracellular nanovolcano recordings. Evolution of the **a** APA, **b** recording duration, and **c** yield of intracellular access during consecutive electroporations for electroporation voltages ranging from 1 to 4 V. **d** Simultaneous recording of action potentials from 13 different channels of a nanovolcano array as obtained after the first electroporation with *V*_EP_ = 2 V (yield of 76.5%)

Biphasic square voltage pulses with amplitudes ranging from 1 to 4 V were applied to nonpatterned nanovolcano arrays covered with spontaneously active cardiomyocyte monolayers to investigate the effect of the electroporation voltage on the yield and quality of the recorded APs. The interval between consecutive electroporations was determined by the time after which none of the microelectrodes of a given device showed intracellular activity anymore (range: ~5 to ~45 min). Figure 3 shows the APAs, recording durations, and yield of successful recordings obtained at increasing electroporation voltages with four different devices. APAs up to 40 mV were successfully recorded, with the highest amplitudes observed at *V*_EP_ = 3 V (cf. Fig. 3a). With each repetition of electroporation, the APAs were further reduced. In the case of *V*_EP_ = 4 V, no intracellular activity was detected after the second repetition, suggesting that this voltage caused irreversible cell damage. Similar to the dependence of the APAs on the electroporation voltage, the recording durations depicted in Fig. 3b suggest that intermediate electroporation voltages (2–3 V) performed best in maximizing periods of intracellular access. With respect to the yield of successful intracellular access following the first electroporation, electroporation voltages ranging from 1 to 3 V induced access in up to 76.5% (*V*_EP_ = 2 V) of the nanovolcanoes (cf. Fig. 3c). Recordings obtained after the first electroporation with *V*_EP_ = 2 V are shown in Fig. 3d. The yield of gaining intracellular access declined with each additional electroporation with *V*_EP_ = 3 V showing the least decrease. Electroporation voltage amplitudes of 4 V produced the lowest yields and failed to initiate successful recordings after two electroporation events. Overall, a *V*_EP_ of 3 V performed best in regard to the APAs, recording durations and yield of intracellular access during repeated electroporations.

### Long-term on-demand recordings with nanovolcano arrays

**Fig. 4:**
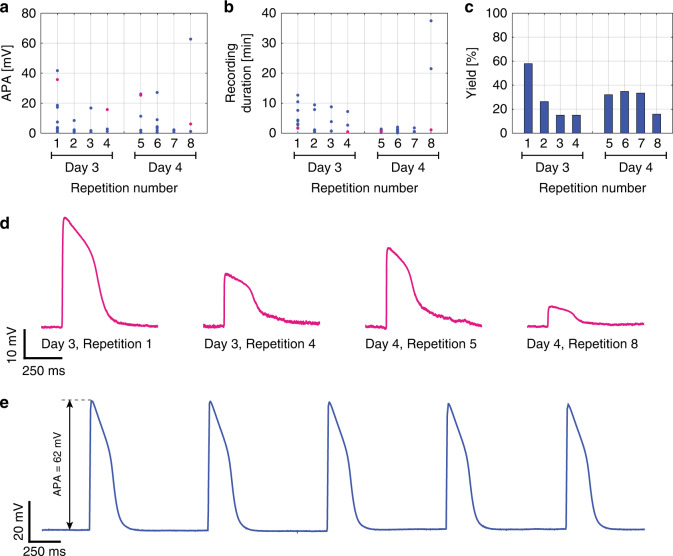
Long-term recording with nonpatterned nanovolcano arrays. Evolution of the **a** APA, **b** recording duration, and **c** yield with two series of electroporations (four each) performed on successive days (*V*_EP_ = 3 V). **d** Action potentials recorded by a single nanovolcano on subsequent days following multiple electroporations. The signals shown correspond to the data points highlighted in magenta in **a** and **b**. **e** Recording of spontaneous action potentials with an exceptionally high amplitude of ~62 mV on day 4 after the preparation underwent 8 electroporations

The electroporation protocols were repeated on the same preparations on successive days to investigate the possibility of using nanovolcano arrays in long-term experiments. The recording characteristics were investigated during four sequential electroporation events (*V*_EP_ = 3 V) that were repeated after 24 h (Fig. 4) in one device. Within the two electroporation sequences performed on different days, the APAs, recording durations and yield decreased in a manner qualitatively similar to that of the data shown above for experiments conducted on the same day (Fig. 3). When comparing the electroporation sequence on day 3 to that of day 4, the APAs showed a slight tendency to recover (sequence 4 vs. sequence 5; Fig. 4a), whereas the recording durations further decreased, with the exception of two recordings in sequence 8 (Fig. 4b). The yield showed a decline similar to that observed in other devices during the electroporation sequence on day 3. On day 4, the success rate remained rather constant during sequential electroporations; i.e., the average success rate did not differ from that on day 3 (Fig. 4c). Figure 4d shows intracellular APs recorded from a single nanovolcano undergoing multiple electroporations on consecutive days (*V*_EP_ = 3 V). The corresponding data points are highlighted in magenta in Fig. 4a, b. Figure 4e depicts intracellular recordings of spontaneous APs as recorded on day 4, with a maximal APA = 62 mV, an average AP duration at 50% repolarization APD_50_ = 166 ± 0.5 ms and a maximal upstroke velocity dV/dt_max_ = 22.8 ± 1.4 %APA/ms (*V*_EP_ = 3 V, repetition 8).

Overall, these results demonstrate that nanovolcanoes paired with electroporation permit on-demand intracellular access in cultures of cardiomyocytes for extended periods of time.

## Discussion

We recently introduced nanovolcanoes as a tool to perform multisite intracellular electrophysiological recordings following spontaneous insertion of arrays of nanodevices into networks of excitable cells^24^. Here, we show that controlled electroporation improves the yield of successful intracellular recordings with nanovolcano arrays and permits long-term on-demand measurements of transmembrane electrical activity in excitable tissues.

### Optimizing the electroporation parameters

Optimal electroporation parameters were derived from an analytical model of the cell–electrode interface based on the electrical properties of nanovolcanoes (measured experimentally) and from the general electrical properties of cell membrane characteristics^31^. The model permitted an estimation of the optimal electroporation signal frequency for efficient perforation of the cell membrane at low voltages. The calculation was based on seal resistances reported previously for similar micro-nanoelectrodes (a few hundred megaohms)^17,32^. This calculation predicted that an electroporation frequency of 10 kHz is optimal to minimize both the electroporation signal attenuation across the electrode–electrolyte interface and the extent of hydrolysis. This prediction was experimentally confirmed, as electroporation voltage amplitudes as low as 600 mV applied at 10 kHz provided successful intracellular access. Furthermore, the use of 10 kHz biphasic square electroporation pulses induced symmetrical and mainly capacitive charge-transfer at the electrode–electrolyte interface^33^, thereby causing charge balance^34^ and preventing the generation of toxic reactive oxygen species^35^. Both of these effects are known to minimize cell damage after electroporation^30^. Electroporation-induced pores in the cell membranes are known to be closely related to pulse duration. While short electroporation pulses (~100 ns) can damage the intracellular compartment without noticeably affecting the cell membrane^36^, longer electroporation pulses induce higher pore densities in the membrane and, therefore, improve the recording performance^37,38^. In this study, an electroporation pulse duration of 100 μs was found to provide an adequate balance between irreversible cell damage induced by electrolysis and a sufficiently high density of electroporation-induced pores.

Even though single electroporation pulses were sometimes found to be sufficient to gain intracellular access with nanovolcanoes, a pulse train with a duration of 1 s was finally adopted to maximize the yield^39^.

Regarding electroporation voltage amplitudes, *V*_EP_ = 3 V provided an adequate balance between sufficiently reducing the junctional resistance and preventing induction of damage to the seal. As reported in the literature^26^, electroporation damages seal resistances, and the poor recording characteristics reported with *V*_EP_ = 4 V are a likely example of this circumstance.

### Performance of functionalized vs. nonfunctionalized nanovolcano tips

The nonconventional ion-beam etching redeposition^40^-based nanofabrication process underlying nanovolcano manufacturing provided sufficient freedom to develop nonpatterned devices, i.e., nanovolcanoes devoid of the intrawall gold layer. Following thiol functionalization, this gold layer was expected to improve the sealing of the cell membrane with the nanovolcano rim. The nonpatterned devices permitted us to investigate the relative importance of thiol functionalization vs. the high curvature of the nanovolcano rim for establishing successful intracellular access. Nonfunctionalized nanopatterned devices were not used on purpose because the Ti–Au–Ti nanoring itself could have affected the results and, hence, biased the study.

The average APAs recorded after the first electroporation amounted to 3.0 ± 2.1 and 6.6 ± 10.1 mV for nonpatterned and patterned nanovolcanoes, respectively. The corresponding yield and recording durations amounted to 73.7%/4.1 ± 3.3 min (nanopatterned) and 63.6%/2.1 ± 2.2 min (nonpatterned; *N* = 14 for all data). Whereas the relatively large variability of values precludes unequivocal conclusions regarding the relative performance of the two types of nanovolcanoes, the overall trend suggests that the geometry of the nanovolcano tip in contact with the cell likely played a dominant role by inducing a tight seal of the cell membrane around the protruding nanostructure^27,28^.

The relatively large variation in APAs, recording durations, and yield among the nanovolcanoes of a given device may be due to several reasons: (i) fabrication-dependent differences in the exact nanostructure of the top of the nanovolcano may have affected the quality of *R*_Seal_, with consequences for all assessed parameters; (ii) the position of individual nanovolcanoes with respect to the monolayer of cardiomyocytes may have resulted in microelectrodes being ideally positioned below a given cell and others being situated at cell–cell borders and, hence, having fewer chances to establish a high-quality seal^41^; and (iii) a few of the nanovolcanoes may have been in contact with noncardiomyocytes (myofibroblasts) that are well known “contaminants” of primary heart cell cultures^42^. These nonexcitable cells are electrotonically coupled to cardiomyocytes and, as a consequence, display passive “APs” with attenuated amplitudes. According to these potential factors inducing variability in recordings, improvements may be made by (i) introducing a polishing procedure to obtain reproducible and seal-promoting flat surfaces of the nanovolcanoes, (ii) controlling the cell positioning with respect to the nanovolcanoes with hydrodynamic traps^43,44^, and (iii) producing cardiomyocyte-only cell cultures by, e.g., fluorescence-activated cell sorting. Moreover, probing signals to investigate the cell–electrode coupling quality might be applied and the results used to compensate APAs according to a calibration curve established beforehand.

Overall, and apart from performing at least similar to, if not better than, patterned nanovolcano devices, nonpatterned nanovolcano devices have the distinct advantage of being based on a simplified preparation procedure that facilitates their production and storage in the case of a potential scale-up.

### Effects of repeated electroporations

Compared with previous results obtained with nanovolcanoes gaining spontaneous intracellular access (maximal APA of 20 mV; maximal yield of 30%; maximal recording duration of 66 min)^24^, the recording success was substantially improved by electroporation. APAs up to 40 mV were frequently observed, with one recording showing an APA of 62 mV, which corresponds to 2/3 of a nonattenuated cardiomyocyte APA^45^. In addition, the yield of nanovolcanoes reporting intracellular electrical activity more than doubled to 76.5%. Similar to the decrease in APAs in the case of nanovolcanoes gaining spontaneous access to the cell interior, the APAs declined after electroporation, indicating that, for both modalities, the cell membrane spanning the opening of the nanovolcano tended to reseal. That electroporation only provides intermittent intracellular access has been previously reported^23,46^. With each consecutive electroporation, the APAs, recording duration and yield showed a decline: when comparing the first with the last (fourth) electroporation (*V*_EP_ = 3 V), the APAs decreased by ~50%, the recording durations by ~30% and the yield by ~75%. The decrease in APAs may be explained by deterioration of the cell–nanovolcano contact and, hence, a decrease in *R*_Seal._ Interestingly, when pausing electroporation for 24 h, both the APAs and yield showed a slight recovery, suggesting partial restitution of the cell–electrode interface.

### On-demand intracellular access for long-term recordings

Compared with our previous work, the possibility of reopening membranes by electroporation increased the apparent overall recording duration by permitting discontinuous on-demand intracellular access over consecutive days. This result suggests that electroporation performed with nanovolcanoes is limited to the small membrane patch defined by the nanovolcano geometry and therefore permits gentle access to the intracellular space without altering cell viability. On average (for *V*_EP_ = 3 V, repetition 1), intracellular access lasted 4.9 ± 3.7 min (*N* = 11; 20 ± 18.2 min, *N* = 3, max. 38 min for *V*_EP_ = 3 V, repetition 8) before the cell membrane resealed. Throughout this period, the APAs declined with time, therefore limiting the use of nanovolcanoes for applications where long-term APA stability is required. Unstable recordings following electroporation are unfortunately a common issue faced by current micro-nanoelectrode arrays. A possible explanation for cell membrane resealing proposed by Spira et al. suggests that the local influx of calcium ions into the cells after electroporation induces exocytosis of intracellular vesicles that patch up the perforated cell membrane^47^. A potential solution to overcome this limitation would be to electroporate holes with larger diameters into the cell membrane, as the self-repair mechanisms are ineffective for larger pore dimensions^48^.

Compared with our previous study where static charge-induced electroporation was suggested as a main penetration mechanism, the recording duration following controlled electroporation is shorter (4.9 ± 3.7 min, *N* = 11; vs. 18.2 ± 21 min, *N* = 16). This result may be explained by assuming that static charge-induced electroporation leads to larger pores, which take more time to reseal. Potentially, nanovolcanoes with higher walls may increase the seal resistance and therefore minimize the calcium influx triggering self-repair mechanisms. Alternatively, longer nonattenuated electroporation pulses across the cell membrane may induce larger holes and improve the recording duration time without triggering adverse electrolysis reactions.

Despite the problems associated with APA attenuation and membrane resealing, repeated measurements on the same site of given preparations are essential advantage over conventional patch-clamp measurements of APs, as it has the potential for screening pharmacological compounds at high throughput and over extended periods of time for cases where AP shapes rather than absolute values of transmembrane voltage changes are of interest. For example, nanovolcanoes permit screening of drug-induced changes in AP duration^24^, which is essential for drug development because prolongation or shortening of APDs is a well-established factor contributing to the precipitation of cardiac arrhythmias. Accordingly, new drug candidates are extensively tested for this unwanted side effect. Because nanovolcano arrays enable quantitative measurements of AP durations, their application in drug development seems accordingly feasible.

### Comparison to other micro-nanotechnology-based electrode arrays granting intracellular access

Micro-nanotechnology-based electrode arrays aim to assess the electrophysiological characteristics of networks of excitable cells over extended periods of time with recording qualities approaching those of standard intracellular measurements. Table 1 compares the respective performance of recently presented micro-nanotechnology-based MEAs. In all experiments, data were collected from primary rat cardiomyocyte cell cultures with intracellular access induced by electro- or opto-poration. By comparison, nanotube-like electrodes composed of IrOx enabled the longest continuous intracellular recordings (~100 min), followed by nanovolcanoes, plasmonic metaelectrodes, nanoneedle,s and micromushrooms. The APAs were largest in the nanovolcanoes, while the maximal yield was best in plasmonic metaelectrodes, followed closely by nanovolcanoes. By contrast to the other technologies, APs measured with nanovolcano arrays did not suffer from high-pass filtering distortion due to the recording system and therefore can be exploited, e.g., for pharmacological studies aiming at detecting side effects of drugs on the AP shape in general and AP duration in particular.

**Table 1:** Summary of features of transmembrane voltage measurements with micro-nanoelectrodes in primary rat cardiomyocyte monolayers

Insertion method	Technology	Max. APA [mV]	Max. duration		Max. yield [%]	Distortion/bandwidth [Hz]
			Single access [min]	Multiple access		
Electroporation	Nanovolcano	62	66^24^	Days	76.5	No/0–8000
	Nanoneedle^17^	20	20	Days	~30	Yes/1–5000
	Micromushroom^19^	6	10	Days	–	Yes/1–10,000
	IrOx nanotube^23^	15	~100	Days	10	Yes/0.01–3000
Optoporation	Plasmonic metaelectrode^50^	5	40	Minutes	85.4	–

### Limitations of the study and outlook

In this study, the electrophysiological recording performance of two different nanovolcano architectures was investigated under different electroporation modalities, with one device being used per condition. This low number does not allow for a systematic statistical comparison of the data. The unequivocal results obtained, however, permit us to draw qualitative conclusions and detect trends when comparing the micro-nanoelectrode devices.

Future developments of nanovolcano arrays may focus on a systematic optimization of the nanovolcano geometry to further optimize its performance. As demonstrated in this work, the protruding nanovolcano geometry is the decisive factor in forming a tight seal at the cell–electrode interface. It is therefore essential to optimize its shape, dimensions, and material composition in a systematic manner to achieve improved recording performance, especially in terms of recording duration. In the literature, hollow nanoelectrodes composed of IrOx showed intracellular recording durations up to 100 min, much longer than those of Au pillars with similar dimensions^23^. As a further improvement, nanovolcanoes may be directly manufactured on integrated CMOS amplifiers in order to maximize signal-to-noise ratios and facilitate the electroporation procedure. This approach would also permit an increase in the density of nanovolcanoes per device, which is now limited by the electrical tracks. Such an approach has been previously described for nanoelectrodes (1024 recording sites with dedicated amplifiers)^17^. Apart from producing larger datasets at minimized experimental cost, a high-resolution array would extend the system capabilities in the context of permitting spatially resolved measurements of electrical activation patterns.

## Conclusion

In this work, we demonstrate that electroporation significantly improves the recording characteristics of transmembrane APs measured with nanovolcano arrays. An analytical model is proposed to predict the electroporation voltage attenuation across the electrode–electrolyte interface and suggests optimal electroporation parameters to perforate cell membranes with low-voltage amplitudes. Electroporation is shown to drastically improve the quality of the electrophysiological recordings reported with nanovolcano arrays in terms of the APAs, recording duration, and yield. The signal quality is shown to be independent of the presence of functionalized nanopatterns incorporated into the tip of the nanovolcano. This finding indicates that the “sharp” geometry of the top of the nanovolcanoes is sufficient to establish optimal seal conditions at the cell–electrode interface. Importantly, electroporation enables on-demand intracellular access during consecutive days. This result suggests that nanovolcanoes have no detrimental effects on cells that they are in contact with, and it opens the possibility to follow electrical activity over periods of time that are relevant for assessing developmental aspects of the preparations under investigation.

## Methods

### Microfabrication

Nanovolcano arrays were manufactured following the nanofabrication process described in detail in our previous work^24^. Briefly, a standard fused silica wafer (100 mm in diameter, 550-μm thick) was covered with a multilayered metal stack by successive evaporations prior to being coated by a 150-nm-thick layer of sputtered SiO_2_. For nanovolcanoes on which a thiol pattern was self-assembled (“patterned nanovolcanoes”), the multilayered stack consisted of Ti–Pt–Ti–Au–Ti–SiO_2_ (10–250–50–50–50–150 nm). For nanovolcano arrays without the self-assembled thiol pattern (“nonpatterned nanovolcanoes”), a stack of Ti–Pt–SiO_2_ (10–250–150 nm) was used instead. Openings 2 μm in diameter were patterned into a 2-μm-thick AZ nLOf 2020 negative photoresist layer (MicroChemicals, Germany) by direct laser writing. Ar^+^ ion-beam etching redeposition was used to manufacture the nanovolcano sidewalls^40^. Next, 3-μm-wide electrical tracks that connected every nanovolcano to its interfacing pad were patterned by photolithography and conventional ion-beam etching. Finally, a 4.5-μm-thick SU8 insulating layer was coated and patterned by direct laser writing, providing 20-μm-diameter openings surrounding each microelectrode of the wafer.

### Device preparation

Nanovolcano arrays were assembled with a glass ring forming the cell culture well using polydimethylsiloxane (Sylgard 184 Silicone Elastomer kit 10:1; Dow Corning, USA) as glue and cured for 2 h at 80 °C. Nanopatterned arrays were sterilized for 30 s by O_2_ plasma (100 W, 650 mTorr; Diener Electronic, Germany) prior to being submerged for 1 h in a 20 mmol/L hexanethiol solution in pure ethanol for self-assembled monolayer formation. After ethanol rinsing, the microelectrode arrays were thoroughly rinsed with sterile deionized water. As previously demonstrated, hexanethiols do not attach to the Pt electrode^26^. For nonpatterned devices, the hexanethiol solution was replaced by pure ethanol. Finally, the substrate was coated with collagen type IV (C5533, Sigma, Germany) before cell seeding. Only new devices were used during this study.

### Isolation and culture of primary rat cardiomyocytes

Primary neonatal rat (Wistar, 1-day old) ventricular cardiomyocytes were isolated and cultured following procedures previously described in detail^49^. Experiments were carried out in compliance with federal guidelines for animal experimentation under license BE27/17 of the Bernese Veterinary Department. In brief, hearts of 4–10 neonatal rats were excised, and ventricles were separated from atria in cold Hank’s balanced saline solution without Ca^2+^ and Mg^2+^ (3-02F29-I, BioConcept, Switzerland) supplemented with trypsin (0.1%; Sigma, T4674) and pancreatin (120 µg/mL; Sigma, P3292). Ventricles were minced using scissors and subjected to 4 or 5 consecutive dissociation cycles in an agitated container maintained at 36 °C. After each cycle, the supernatant was removed and stored on ice. New dissociation solution was added to the remaining tissue pieces, and the next dissociation cycle was started. The dissociated cells were spun down and resuspended in M199 medium (M7653, Sigma) supplemented with penicillin (20 U/mL; Sigma, P7794), vitamin B_12_ (2 µg/mL; Sigma, V2876), vitamin C (18 µmol/L; Sigma A-4544), epinephrine (10 µM; Sigma, E4250), bromodeoxyuridin (100 µmol/L; Sigma, B-5002), L-glutamine (680 µM; Sigma, G7513), and neonatal calf serum (S 0125, Biochrom, Bioswisstec, Switzerland). Cardiomyocytes were purified by differential preplating in cell-culture flasks. After 2 h, the supernatant containing mostly cardiomyocytes was removed from the flasks, and the cell number was determined using a hemocytometer. Dilution was adjusted to achieve a seeding density of ~3500 cardiomyocytes per mm^2^. Preparations were incubated for 24 h at 36 °C in a humidified atmosphere containing 0.8% CO_2_. After 24 h, the cell-culture medium was replaced, and the serum concentration was lowered from 10 to 5%. Thereafter, the cell-culture medium was replaced every other day.

### Electroporation procedure

Prior to the first electroporation, a control recording was performed to guarantee the absence of intracellular activity due to static charge-induced electroporation^24^. Thereafter, before initiating the electrophysiological recordings, biphasic voltage pulses with a period of 100 μs and an amplitude ranging from 1 to 4 V with respect to the four reference electrodes integrated in the device were simultaneously applied to every channel of the device using an A-M Systems Model 2100 pulse stimulator (A-M Systems, USA) for an overall duration of 1 s.

### Electrophysiology

Electrophysiological experiments were started 48 h after cell seeding. At this time, the cardiomyocytes had formed a uniform cell monolayer that exhibited spontaneous synchronized electrical activity, and contamination by proliferating noncardiomyocytes was still moderate. Experiments were performed in a dry incubator (37 °C; 0.8% CO_2_) using a DC-coupled HS-36 headstage (*R*_in_ = 1 TΩ, *C*_in_ = 2 pF; bandwidth = 0–8 kHz) together with a Digital Lynx SX acquisition system (Neuralynx, USA). Preparations were covered with a lid to prevent evaporation.

### Data analysis

A custom-made MATLAB2016a (MathWorks, USA) script was developed to extract the electrophysiological parameters of interest from the recorded signals (APA, APD_50_, and dV/dt_max_) as well as the recording duration per channel and intracellular yield per device. Every electrical trace analyzed and presented in this work corresponds to the nonfiltered raw data.

To objectively define the duration of intracellular recording per channel, an AP was considered intracellular until its amplitude decreased below 250 μV or until the maximum of the first derivative thereof became smaller than three times the absolute value of its minimum, which is typical for extracellular recordings.

Recordings from channels showing saturation were excluded from the analysis. Saturation occurring in a few channels was likely the result of the combination of the relatively large impedance of the nanovolcanoes with the DC-coupled headstage of the recording system.

Values are given as the mean ± standard deviation (mean ± SD) throughout the manuscript.

## Supplementary information

Supplementary Information
