# Supplementary material for: Nanovolcano microelectrode arrays: toward long-term on-demand registration of transmembrane action potentials by controlled electroporation
Source: Microsyst Nanoeng. 2020 Aug 24;6:67. doi: 10.1038/s41378-020-0178-7 (PMC8433144; doi:10.1038/s41378-020-0178-7)
Supplement: Supplementary file 1 — Supplementary Information [file 41378_2020_178_MOESM1_ESM.pdf]

Supplementary Information

Nanovolcano-microelectrode-arrays: Towards long-term on-demand registration of transmembrane action potentials by controlled electroporation

B.X.E. Desbiolles[1]*, E. de Coulon[2]*, N. Maino[1], A. Bertsch[1], S. Rohr[2&], and P. Renaud[1&]

Table of content

1 Layout of the microelectrode arrays 2
2 Electroporation modelling 3
2.1 Numerical values of the equivalent electrical circuit of the cell-electrode interface. 3
2.2 Analytical expression of V_j as a function of V_{EP} and R_{seal} 3
3 Evolution of the signal amplitude with time after electroporation 5
4 Neonatal rat cardiomyocyte monolayer 6
5 References 7

[1] Laboratory of Microsystems LMIS4, Ecole Polytechnique Fédérale de Lausanne, Lausanne, Switzerland.

[2] Group Rohr, Department of Physiology, University of Bern, Bern, Switzerland

* Shared first authorship

[&] Shared last authorship

Correspondence: Benoit Xavier Emmanuel Desbiolles (benoit.desbiolles@epfl.ch)

1 Layout of the microelectrode arrays

Figure S1 depicts the microelectrode array layout used in this study. The array was composed of 32 recording microelectrodes (28 nanovolcanoes and 4 planar Pt electrodes with a diameter of 40 µm), 2 stimulation dipoles, and 3 reference electrodes. The microelectrodes are separated by a pitch of 500 µm.

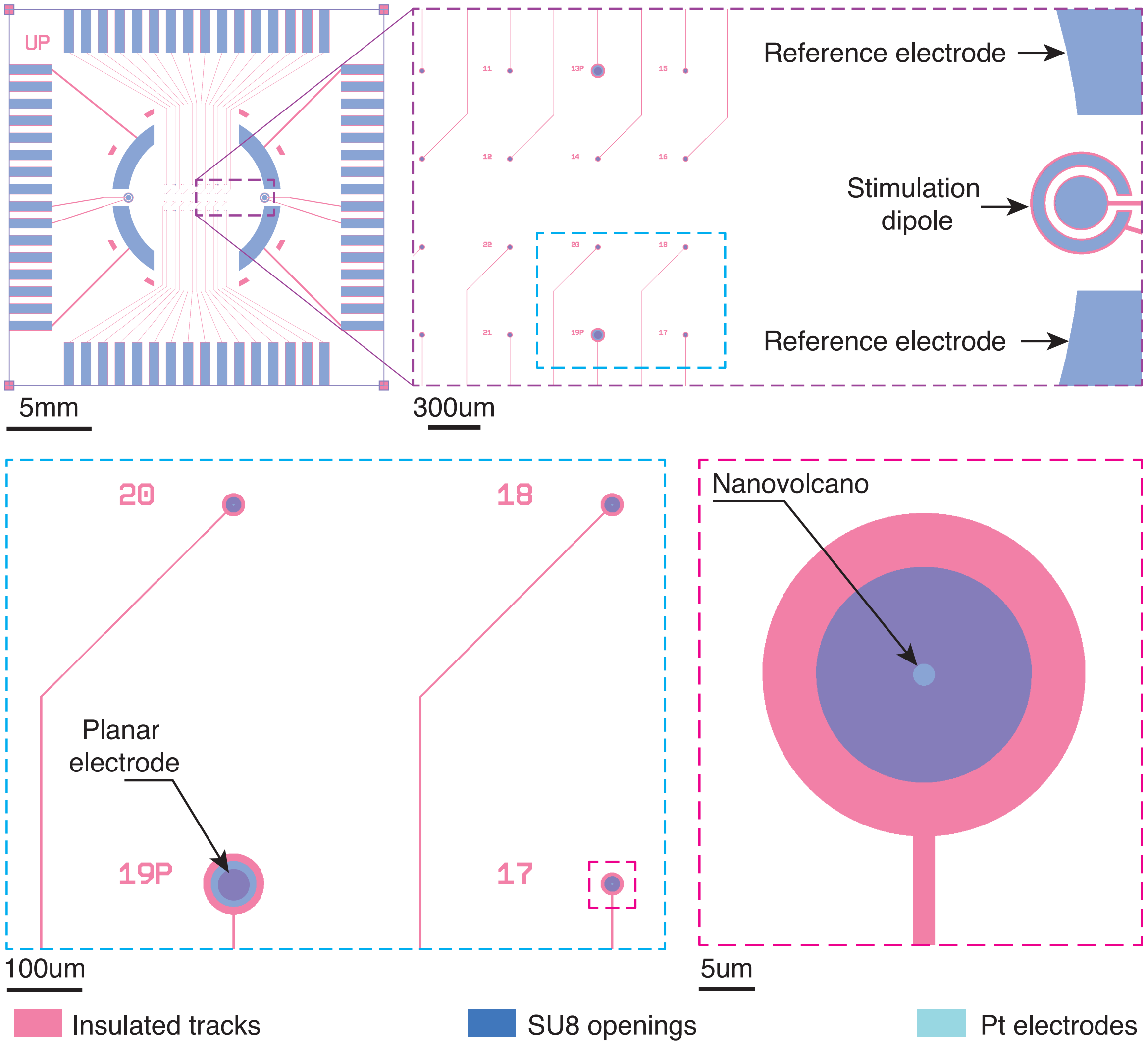

Figure S1: *Layout of the microelectrode array.* Electrically conductive tracks (pink) connect each electrode to the interfacing pads of the chip. The inserts show an enlarged view of the recording / stimulation electrodes (top right panel), the planar electrodes used as control recording sites (bottom left panel), and the nanovolcano (bottom right panel).

2 Electroporation modelling

This section gives an overview of the analytical computations used to model the cell-electrode interface during electroporation.

2.1 Numerical values of the equivalent electrical circuit of the cell-electrode interface

The values for each element forming the cell-electrode interface equivalent circuit are summarized in Table S1. Components related to the electrode-electrolyte interface were experimentally measured (i.e. R_{CT}, CPE_{DL}, and C_{Stray}) whereas the junctional cell membrane capacitance, C_j, and resistance, R_j, were taken from the literature.[1]

Table S1: Values of the elements composing the electrical equivalent circuit of the electrode-electrolyte interface (for schematic cf. main article)

Element	Value	Reference
R_{CT}	1.3 GΩ	[2]
C_{DL}, n	18.6 pF, 0.85	[2]
C_{Stray}	0.3 pF	[2]
C_j	30 fF	[1]
R_j	47 GΩ	[1]

2.2 Analytical expression of V_j as a function of V_{EP} and R_{seal}

The attenuation of V_{EP}, applied at the device terminals toward the junctional space, is defined by the voltage divider between the electrode impedance ($Z_e = CPE_{DL} \,||\, R_{CT}$) and the impedance representing the rest of the cell-electrode interface ($Z_{ce} = R_{seal} \,||\, R_j \,||\, C_j$). In the frequency domain, Z_e can be expressed as:

$$Z_e = \frac{R_{CT}}{R_{CT}C_{DL}(j\omega)^n + 1}$$

Similarly, Z_{ce} can be defined as:

$$Z_{ce} = \frac{R_j R_{seal}}{(R_j C_j j\omega + 1)(R_{seal} + \frac{R_j}{R_j C_j j\omega + 1})}$$

V_j can subsequently be defined as:

$$V_j = \frac{Z_{ce}}{Z_{ce} + Z_e} V_{EP}$$

This expression was used to analytically determine the ratio V_j / V_{EP} presented in the main article Figure 1-B.

3 Evolution of the signal amplitude with time after electroporation

Figure S2 presents a typical example of the decay of APAs after electroporation. A relatively rapid initial decay is followed, after ~ 2 minutes, by a slower decay. After 8 minutes, signals with typical AP shapes are superseded by signals showing both intracellular and extracellular signal characteristics which is likely explained by progressive re-sealing of the cell.

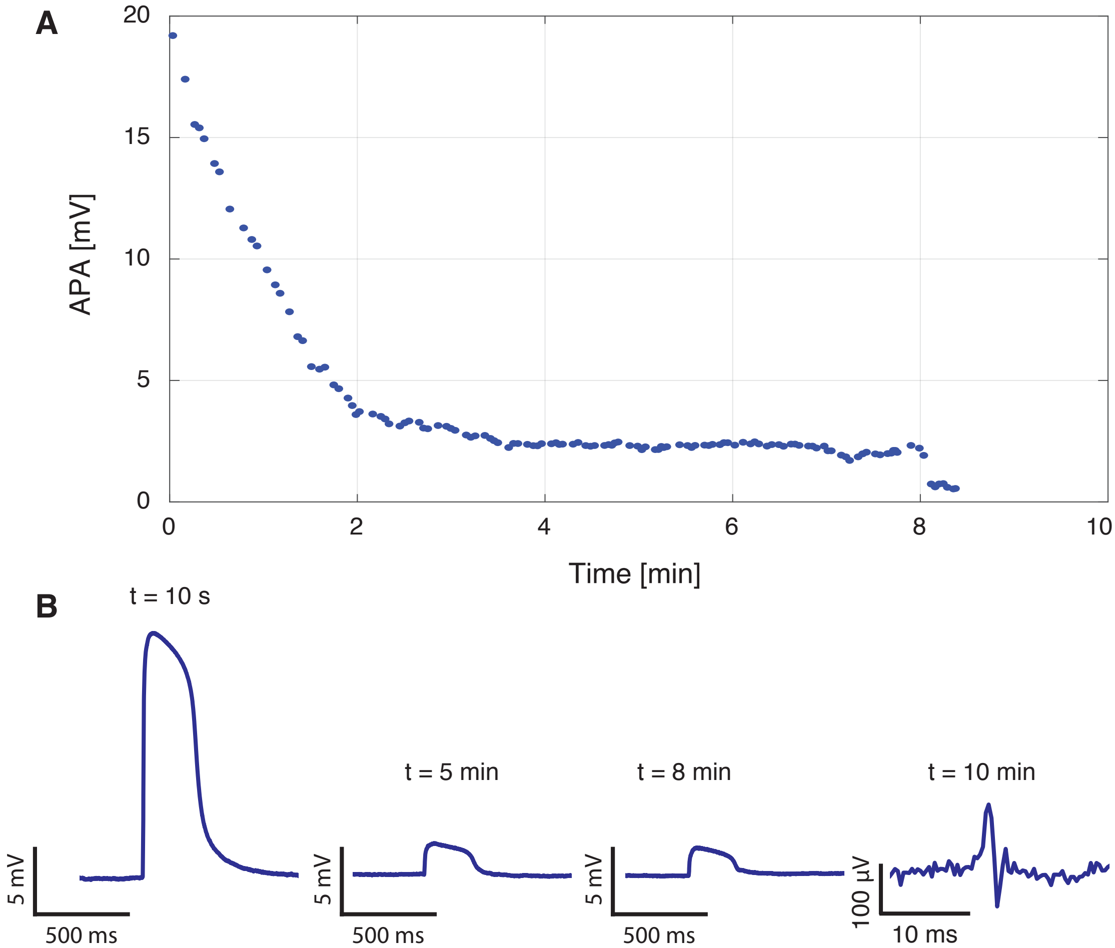

Figure S2: *APA temporal evolution after electroporation.* A) Typical time course of the decline of the action potential amplitude (APA) as reported by a single nanovolcano after electroporation (V_{EP} = 1V, repetition 1). B) Electrical trace of the corresponding action potentials at different time points.

4 Neonatal rat cardiomyocyte monolayer cultured on a nanovolcano array

Figure S3 shows a representative phase contrast image of a typical 1-day-old culture of neonatal rat cardiomyocyte monolayer grown on a nanovolcano array. Cell morphologies remained unchanged after multiple electroporation events.

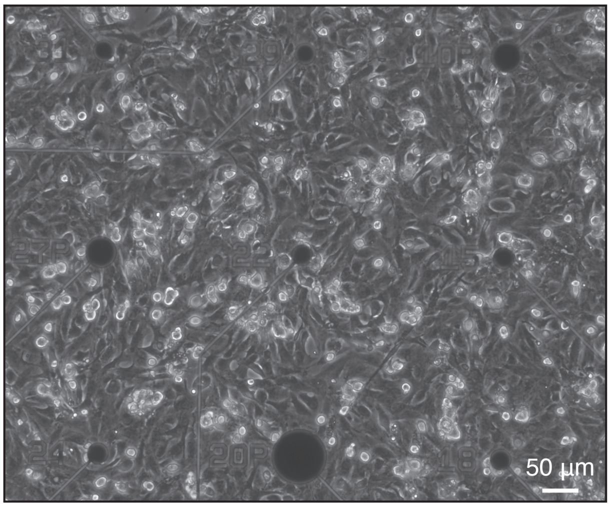

Figure S3: Phase contrast image of a 1-day-old monolayer culture of primary neonatal rat cardiomyocyte grown on a nanovolcano array.

5 References

1. R. Milo and R. Phillips, *Cell Biology by the numbers*, (2015).
2. B. X. E. Desbiolles, E. De Coulon, A. Bertsch, S. Rohr and P. Renaud, Intracellular Recording of Cardiomyocyte Action Potentials with Nanopatterned Volcano-Shaped Microelectrode Arrays, *Nano Letters*, 19, 6173–6181, **2019**.
